# Supplementary material for: Content-rich biological network constructed by mining PubMed abstracts
Source: BMC Bioinformatics. 2004 Oct 8;5:147. doi: 10.1186/1471-2105-5-147 (PMC528731; doi:10.1186/1471-2105-5-147)
Supplement: Additional File 2 — The original results of the above study (non-essential files are deleted to keep the file size under the limit set by BMC bioinformatics). [file 1471-2105-5-147-S2.bz2 › chilibotAdditionalFile2/dip50/154ID7630397E551/html/CDK2_CCNA2.html]

 


 **CDK2** and **CCNA2** 
  
Found 650 abstracts in PubMed, retrieved 50.  
 

 What does Google say? 
 PDF only 
| .edu only 

---

**Interactive relationship** (e.g. stimulation, inhibition, etc)

**Inhibitory relationship**- The inhibition of cyclin A  [ **CCNA2** ]  gene expression is associated with a block in cyclin E  **cdk2**  activity and phosphorylation of both the retinoblastoma protein and p107.  Ref: 12869629 Mol Pharmacol, 2003
- In addition, rapamycin treatment inhibited cyclin dependent kinase 2  [ **CDK2** ]  activity by preventing up regulation of cyclin E and cyclin A  [ **CCNA2** ] .  Ref: 12393642 Blood, 2003
- Western blot analysis also shows that DHA greatly reduces the level of cyclin A  [ **CCNA2** ] , while increasing the level of p21 WAF1, a cellular inhibitor of cyclin A  [ **CCNA2** ]  cyclin dependent kinase 2  [ **CDK2** ]  activity.  Ref: 12492401 Biochem J< MedlineTA>Biochem J, 2003
**Neutral relationship**- Cyclin A  [ **CCNA2** ]  binds to  **CDK2**  and plays critical roles when cells proliferate.  Ref: 12395374 Hum Pathol, 2002
- Arg Lys is recognized by  **CDK2** , while the cyclin A  [ **CCNA2** ]  component of the complex contains a hydrophobic site that binds Arg Lys.X.  Ref: 12501191 Biochemistry, 2002
- Cyclin A  [ **CCNA2** ]  was co expressed with  **cdk2**  in the proliferating cells, ie those showing positive Ki 67 staining.  Ref: 12533834 J Pathol, 2003
- This is a novel mechanism of TGF beta1 inhibition requiring early G 1 induction and stabilization of p21 protein, which binds to and inhibits cyclin E  **CDK2**  and cyclin A  [ **CCNA2** ]   **CDK2**  kinase activity rather than direct modulation of cyclin or CDK protein levels as seen in other systems.  Ref: 12810668 Cancer Res, 2003
- However,  **CDK2**  bound by the truncated cyclin A2  [ **CCNA2** ]  is located in the cytoplasm in contrast to  **CDK2**  bound to full length cyclin A2  [ **CCNA2** ] , which is in the nucleus.  Ref: 12646244 Biochem Biophys Res Commun, 2003
- Cyclin A  [ **CCNA2** ]  is expressed during the S phase and cyclin A  [ **CCNA2** ]   **CDK2**  is important for a normal rate of DNA elongation.  Ref: 12653640 Biochem Soc Trans, 2003
- The truncated cyclin A2  [ **CCNA2** ]  binds to and activates the cyclin dependent kinase 2  [ **CDK2** ]   **CDK2** .  Ref: 12646244 Biochem Biophys Res Commun, 2003
- The decrease in  **CDK2**  kinase activity was the result of increased p21 association with cyclin A  [ **CCNA2** ]   **CDK2**  and cyclin E  **CDK2** .  Ref: 12810668 Cancer Res, 2003
- However, in both cell types Cdk 2  [ **CDK2** ]  activity associated with cyclin E and cyclin A  [ **CCNA2** ]  showed an increase only when the cells transited from G 1 into S phase.  Ref: 12704643 J Cell Physiol, 2003

**Non-interactive relationship** (e.g. studied together, co-existance, homology, etc.)

- METHODS The molecular expressions of Rb, cyclin A  [ **CCNA2** ] , cyclin E,  **CDK2**  and CDC2 involved in S phase progression were investigated by westernblot in L 2 and Br1 3pr 1 cells.  Ref: 12600080 Hua Xi Yi Ke Da Xue Xue Bao, 2001
- Expression of cyclin A  [ **CCNA2** ] , cyclin E and  **cdk2**  was examined immunohistochemically in 144 cases of primary non small cell lung carcinoma to evaluate their prognostic value.  Ref: 12533834 J Pathol, 2003
- A microscopic study of functional disorder order folding transitions coupled to binding is performed for the p27 protein, which derives a kinetic advantage from the intrinsically disordered unbound form on binding with the phosphorylated cyclin A  [ **CCNA2** ]  cyclin dependent kinase 2  [ **CDK2** ]   **Cdk2**  complex.  Ref: 12697905 Proc Natl Acad Sci U S A, 2003
- When we examined the effects of this drug on A498 cells, arsenic trioxide decreased the levels of  **CDK2** , CDK6, cyclin D1, cyclin E, and cyclin A  [ **CCNA2** ]  proteins.  Ref: 12480548 Biochem Biophys Res Commun, 2003
- Adriamycin induced G1 S arrest seems to be realized via cyclin cdk complexes activity independent way involving antiproliferative targets downstream of cyclin E  **cdk2**  and cyclin A  [ **CCNA2** ]   **cdk2**  complexes.  Ref: 12722479 Tsitologiia, 2003
- To clarify the role of cyclin A  [ **CCNA2** ]  in multistage colorectal neoplasms, cyclin A  [ **CCNA2** ] ,  **CDK2** , and Ki67 were immunohistochemically stained in 22 normal mucosa, 9 hyperplastic polyps, 61 adenomas, 197 primary carcinomas, 21 lymph node metastases, and 10 hepatic metastases.  Ref: 12395374 Hum Pathol, 2002
- Arsenic increased expression of the P21 protein and decreased levels of cyclin A  [ **CCNA2** ] , cyclin B1 and cyclin D1, but expression of  **CDK2** , CDK4, CDK6, and cyclin E were not affected.  Ref: 12883267 Pancreas, 2003
- Cyclin E  **CDK2**  and cyclin A  [ **CCNA2** ]   **CDK2** , together with DBF4 CDC7, phosphorylate several components of the prereplication complex and replication machinery.  Ref: 12851482 Cell Cycle< MedlineTA>Cell Cycle, 2003
- Monensin decreased the levels of  **CDK2** , CDK6, cdc2, cyclin A  [ **CCNA2** ] , cyclin B1, cyclin D1 and cyclin E proteins but did not alter CDK4 protein.  Ref: 12792794 Int J Oncol, 2003
- Following DNA damage, the p53 dependent induction of p21CIP1 regulates cyclin E  **Cdk2**  and cyclin A  [ **CCNA2** ]   **Cdk2**  complexes both of which phosphorylate pRB, leading to E2F mediated activation.  Ref: 12613199 Cancer Treat Res, 2003
- Progression through S phase of the eukaryotic cell cycle is regulated by the action of the cyclin dependent protein kinase 2  **CDK2**  in association with cyclin A  [ **CCNA2** ] .  Ref: 12501191 Biochemistry, 2002
- Positive staining for cyclin D1, cyclin E, cyclin A  [ **CCNA2** ] , cyclin B1,  **cdk2** , cdk4, cdc2, p53, p21, and p27 was observed in 63%, 66%, 31%, 32%, 51%, 77%, 71%, 43%, 35%, and 60% of the 82 carcinomas, respectively.  Ref: 12792921 Hum Pathol, 2003
- The observations have implications for the further identification of physiological substrates of  **CDK2**  cyclin A  [ **CCNA2** ]  and the design of specific inhibitors.  Ref: 12501191 Biochemistry, 2002
- Pluripotent cell division cycles are driven by ectopic  **Cdk2** , cyclin A  [ **CCNA2** ]  E and E2F activities.  Ref: 12447695 Oncogene, 2002
- When we examined the effects of this drug on SNU C1 cells, monensin decreased the levels of  **CDK2** , CDK4, CDK6, cyclin D1 and cyclin A  [ **CCNA2** ]  proteins.  Ref: 12527937 Int J Oncol, 2003
- it delayed the activity of the cyclin A  [ **CCNA2** ]   **cdk2**  complex to 34h.  Ref: 12876382 Cell Physiol Biochem, 2003
- Western blotting analysis revealed that the protein levels of cyclin A  [ **CCNA2** ] , cyclin E, and cyclin dependent kinase CDK 2  [ **CDK2** ]  but not cyclin D1 and CDK4 decreased after progesterone treatment, but those of CDK inhibitory proteins, p21 and p27, increased.  Ref: 12810531 Endocrinology< MedlineTA>Endocrinology, 2003
- Prenatal ethanol exposure produced a significant down regulation of  **CDK2**  cyclin A  [ **CCNA2** ]  expression.  Ref: 12480138 Brain Res Dev Brain Res, 2002
- B Myb and cyclin A2  [ **CCNA2** ]  formed mutually exclusive complexes with p107, and B Myb enhanced the activity of co transfected cyclin E kinase activity, implying that B Myb affects the cell cycle by preventing sequestration of active cyclin  **cdk2**  complexes.  Ref: 12439743 Oncogene, 2002
- Analysis of the expression of cell cycle related proteins after the addition of catechin showed that the cyclin dependent kinase 2  [ **CDK2** ]  and the cdk4 proteins were decreased after administration, the expression of cyclin A  [ **CCNA2** ]  protein was increased at 24 h after administration the expression of the cyclin D1 and cyclin E proteins was unchanged.  Ref: 12429981 Int J Oncol, 2002
- We present evidence indicating that  **Cdk2**  cyclin E complexes must become highly concentrated within nuclei to support replication and find that cyclin A  [ **CCNA2** ]  can trigger replication at much lower intranuclear concentrations.  Ref: 12475960 Mol Biol Cell, 2002
- These cells display precocious  **Cdk2** , cyclin A  [ **CCNA2** ]  and cyclin E kinase activities that are conspicuously cell cycle independent.  Ref: 12447695 Oncogene, 2002
- The roles of cyclin A  [ **CCNA2** ]  E  **CDK2**  complexes in driving replication, their relationship with other regulators of S phase, and their role in keeping replication to only once per cell cycle will be discussed.  Ref: 12851482 Cell Cycle< MedlineTA>Cell Cycle, 2002
- PG inhibited cyclin E,  **cdk2** , p27 and p21, the induction of the cyclin A  [ **CCNA2** ]   **cdk2**  and cyclin E  **cdk2**  kinase activity, and the phosphorylation of Rb in leukaemic Jurkat cells.  Ref: 12647787 Histol Histopathol, 2003
- When we examined the effects of this drug on ACHN cells, monensin decreased the levels of  **CDK2** , CDK6, cdc2, cyclin A  [ **CCNA2** ]  and cyclin B1 proteins.  Ref: 12632079 Int J Oncol, 2003
- Specificity determinants of recruitment peptides bound to phospho  **CDK2**  cyclin A  [ **CCNA2** ] .  Ref: 12501191 Biochemistry, 2002
- To determine additional sequence specificity motifs around the RXL sequence, we have performed X ray crystallographic studies at 2.3 A resolution and isothermal calorimetry measurements on complexes of phospho  **CDK2**  cyclin A  [ **CCNA2** ]  with a recruitment peptide derived from E2F1 and with shorter 11 mer peptides from p53, pRb, p27, E2F1, and p107.  Ref: 12501191 Biochemistry, 2002
- Cyclin A  [ **CCNA2** ]   **cdk2**  appears to associate with NF Y both in vitro and in vivo.  Ref: 12857729 J Biol ChemJ Biol Chem, 2002
- In conclusion, we have adopted an experimental system enabling us to study the signaling pathways in cells grown under anchorage independent conditions and have identified matrix independent activation of PI 3 kinase and Stat3 signaling functions, as well as the PI 3 kinase dependent increase of cyclin A  [ **CCNA2** ]  associated  **Cdk2**  kinase activity, to be critical for the Ros PTK induced anchorage independent growth.  Ref: 12646574 J Biol Chem, 2003
- In Ct treated rats, the expression of  **CDK2**  and its activator, cyclin A  [ **CCNA2** ] , paralleled the pattern of granule cell proliferation.  Ref: 12480138 Brain Res Dev Brain Res, 2002
- Similarly, levels of the p21 WAF1 CIP1 protein that is associated with the Cyclin A  [ **CCNA2** ]   **Cdk2**  complex increased, and these increases correlated well with the down regulation of Cyclin A  [ **CCNA2** ]   **Cdk2**  activity.  Ref: 12819186 Carcinogenesis< MedlineTA>Carcinogenesis, 2002
- Elevated cyclin A  [ **CCNA2** ]  not only was linked with elevated  **CDK2**  in primary cancers, but also was associated with increased Ki67 in both adenomas and primary carcinomas.  Ref: 12395374 Hum Pathol, 2002
- In vitro kinase assays demonstrated a corresponding massive activation of the cyclin A  [ **CCNA2** ]  regulated cyclin dependent kinase 2  [ **CDK2** ]   **cdk2**  but not of  **cdk2**  protein level in muscle homogenates after 1.5 days of muscle loading.  Ref: 12819222 J Appl Physiol< MedlineTA>J Appl Physiol, 2002
- Thus, PD selectively elevates p21 WAF1 CIP1 levels and thereby arrests the cell cycle at G1 S phase by down regulating Cyclin A  [ **CCNA2** ]   **Cdk2**  activity.  Ref: 12819186 Carcinogenesis< MedlineTA>Carcinogenesis, 2002
- Furthermore, cyclin A  [ **CCNA2** ]  CDK1 2, cyclin B CDK1 2 and cyclin E  **CDK2**  complexes can phosphorylate multiple p73 isoforms in vitro at threonine 86 T86.  Ref: 12676926 J Biol Chem< MedlineTA>J Biol Chem, 2002
- Increased p27 and p21 levels coincided with reduced activities of cell cycle dependent kinases cdk such as  **cdk2**  and cyclin A  [ **CCNA2** ]  protein kinases 24 h after TRO treatment.  Ref: 12595159 Toxicol Lett, 2003
- When cells were synchronized in S phase with aphidicolin and then released, activation of Delta MEKK3 ER\* resulted in the up regulation of p21 CIP1 and a pronounced inhibition of cyclin A  [ **CCNA2** ]   **CDK2**  and cyclin B1 CDK1 kinase activity.  Ref: 12444545 Oncogene, 2002
- Furthermore, YA protein is phosphorylated in parallel with a cell cycle dependent activation of  **cdk2**  kinase and cyclin A  [ **CCNA2** ]  expression.  Ref: 12857729 J Biol ChemJ Biol Chem, 2002
- This effect is specific for these complexes since SET did not inhibit either cyclin A  [ **CCNA2** ]   **CDK2**  or cyclin E  **CDK2**  complexes.  Ref: 12407107 J Biol Chem, 2003
- Panaxadiol selectively inhibits Cyclin A  [ **CCNA2** ]  associated  **Cdk2**  activity by elevating p21WAF1 CIP1 protein levels in mammalian cells.  Ref: 12819186 Carcinogenesis< MedlineTA>Carcinogenesis, 2003
- Matrix independent activation of phosphatidylinositol 3 kinase, Stat3, and cyclin A  [ **CCNA2** ]  associated  **Cdk2**  Is essential for anchorage independent growth of v Ros transformed chicken embryo fibroblasts.  Ref: 12646574 J Biol Chem, 2003
- PD treated cells were arrested at G1 S phase, which coincided well with decreases in Cyclin A  [ **CCNA2** ]   **Cdk2**  activity, but not in Cyclin E  **Cdk2**  and Cdc2 activities.  Ref: 12819186 Carcinogenesis< MedlineTA>Carcinogenesis, 2003
- Overexpression of the replication initiation factors Cdt1 and Cdc6 along with cyclin A  [ **CCNA2** ]   **cdk2**  promotes rereplication in human cancer cells with inactive p53 but not in cells with functional p53.  Ref: 12718885 Mol Cell, 2003
- Immunoprecipitation studies demonstrated that Sp1 interacts with cyclin E, cyclin A  [ **CCNA2** ]  and cyclin dependent kinase 2  [ **CDK2** ]  during the S phase.  Ref: 12794070 J Biol Chem< MedlineTA>J Biol Chem, 2003
- Expression level of cyclin A  [ **CCNA2** ]  was similar to p27 and p57 but not to  **CDK2**  and increased to a peak level at day 2.  Ref: 12526100 Exp Mol Med, 2002
- **CDK2**  cyclin A  [ **CCNA2** ]  phosphorylates numerous substrates.  Ref: 12501191 Biochemistry, 2002
- UR2 infected CEF grown in nonadherent conditions displayed faster cell cycle progression than the control or the UR2 mutant infected cells, and this appeared to correlate with a PI 3 kinase dependent increase in cyclin A  [ **CCNA2** ]  associated  **Cdk2**  activity.  Ref: 12646574 J Biol Chem, 2003
